# Structural disruption of the blood–brain barrier in repetitive primary blast injury

**DOI:** 10.1186/s12987-020-00231-2

**Published:** 2021-01-07

**Authors:** Gozde Uzunalli, Seth Herr, Alexandra M. Dieterly, Riyi Shi, L. Tiffany Lyle

**Affiliations:** 1 Department of Comparative Pathobiology, Purdue University College of Veterinary Medicine, West Lafayette, IN USA; 2 Department of Basic Medical Sciences, Purdue University College of Veterinary Medicine, West Lafayette, IN USA; 3 Purdue University Weldon School of Biomedical Engineering, West Lafayette, IN USA; 4 Center for Cancer Research, Purdue University, West Lafayette, IN USA; 5 Center for Comparative Translational Research, Purdue University, West Lafayette, IN USA

**Keywords:** Blast-induced traumatic brain injury, Repetitive injury, Blood–brain barrier, Pericytes, CD13, Desmin, Neurotrauma

## Abstract

**Background:**

Blast-induced traumatic brain injury (bTBI) is a growing health concern due to the increased use of low-cost improvised explosive devices in modern warfare. Mild blast exposures are common amongst military personnel; however, these women and men typically do not have adequate recovery time from their injuries due to the transient nature of behavioral symptoms. bTBI has been linked to heterogeneous neuropathology, including brain edema, neuronal degeneration and cognitive abnormalities depending on the intensity of blast overpressure and frequency. Recent studies have reported heterogeneity in blood–brain barrier (BBB) permeability following blast injury. There still remains a limited understanding of the pathologic changes in the BBB following primary blast injuries. In this study, our goal was to elucidate the pathologic pattern of BBB damage through structural analysis following single and repetitive blast injury using a clinically relevant rat model of bTBI.

**Methods:**

A validated, open-ended shock tube model was used to deliver single or repetitive primary blast waves. The pathology of the BBB was assessed using immunofluorescence and immunohistochemistry assays. All data were analyzed using the one-way ANOVA test.

**Results:**

We have demonstrated that exposure to repetitive blast injury affects the desmin-positive and CD13-positive subpopulations of pericytes in the BBB. Changes in astrocytes and microglia were also detected.

**Conclusion:**

This study provides analysis of the BBB components after repetitive blast injury. These results will be critical as preventative and therapeutic strategies are established for veterans recovering from blast-induced traumatic brain injury.
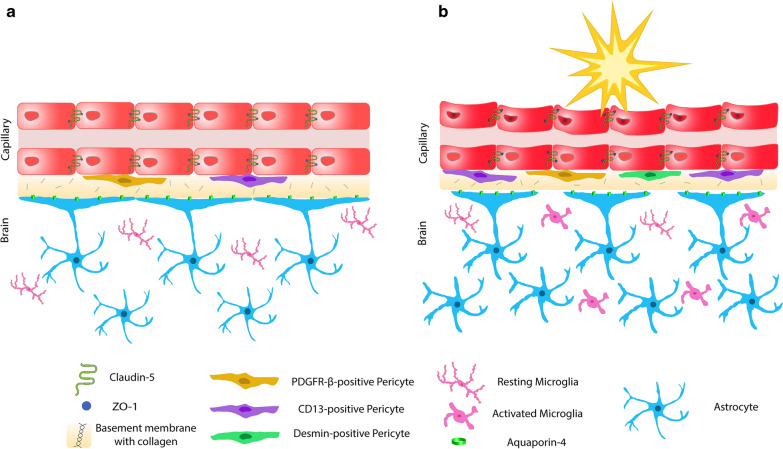

## Introduction

There has been a surge in blast-induced traumatic brain injury (bTBI) among veterans due to the frequent use of low-cost improvised explosive devices (IEDs) in modern warfare. Approximately 1 in 5 soldiers have suffered from traumatic brain injury, and of these, 52% are due to blast initiated by IEDs [1]. Thus, bTBI is often called the signature injury of modern warfare, as it is the most common form of head trauma in theatre. Recent evidence highlights that brain injury is directly associated with blast shock waves, shown to play a significant role in both morbidity and mortality [2–4]. Often in warfare, soldiers exposed to bTBI pass medical evaluations because of transient or mild behavioral symptoms, and they return to duty without adequate recovery time and can be exposed to additional mild blast waves. Studies have shown that blast injuries, especially repetitive blast injuries, lead to the progression of neuropathology in both animal models and human patients [5, 6]. Among wounded soldiers, bTBI has been linked to numerous neurodegenerative diseases and neurologic comorbidities such as post-traumatic stress disorder (PTSD), depression, Parkinson’s disease (PD), and Alzheimer’s disease (AD) [1, 7–9]. Unfortunately, while it is well established that bTBI can lead to neurodegenerative diseases, the mechanism is unclear. This is mainly due to the poor understanding of the bTBI pathologies, which are critical to developing effective diagnostic and treatment strategies to intervene and deter post-bTBI neurodegeneration. While a detailed mechanism of bTBI damage is not clear, strong evidence has emerged to support a key pathological role of the blood–brain barrier (BBB). The BBB is the tightest and most robust barrier in the body. This impermeable barrier limits macromolecule entry into the brain to maintain the physiological environment for brain function. BBB damage associated with bTBI contributes to cerebrovascular injuries such as hemorrhage, hematoma, vasospasm, and brain edema [7]. In addition, BBB disruption may allow an influx of blood-derived neurotoxins, including unwanted cells or microbial pathogens, leading to chronic inflammatory and immune responses [10].

It is well established that BBB damage has been implicated and well recognized to play a critical pathological role in leading neurodegenerative diseases. In the case of AD, persistent vascular permeability and inflammation in the brain have been correlated with rapid disease onset and severity [11]. Similarly, BBB damage has been found in the striatum of PD patients, a brain region critical to PD pathology.

Although post-bTBI BBB damage has been examined, previous studies have concentrated on functional leakage, and not investigated the potential cellular or structural pathology in the BBB that lead to or cause its permeability. Consequently, little is known about the cellular and structural components of BBB damage post-bTBI. Therefore, it is critically important to elucidate the details of the underlying pathology and the possible mechanism of post-TBI BBB damage [12].

Our lab has an established model of mild blast injury that recapitulates the clinical phenomenon of mild bTBI. Specifically, rats subjected to mild bTBI displayed no clear acute motor deficits, which is in agreement with clinical observations. Despite the lack of behavioral changes, our model shows conspicuous BBB leakage on the ventral region of the brain after a single mild bTBI [13]. Furthermore, we have noted initial PD-like pathologies in the brain basal ganglia following mild bTBI [14]. Taken together, we have established a clinically relevant bTBI model that is suitable for our current investigation of BBB alterations. We aimed to utilize this model to elucidate the details of BBB damage through structural analysis. These efforts will enhance our ability to diagnose, and more importantly, to establish reparative strategies to delay or even prevent post-bTBI neurodegenerative diseases.

## Materials and methods

### Primary blast-induced traumatic brain injury animal model

In vivo experiments were carried out with adult male Sprague Dawley rats weighing approximately 300 g. Rats were maintained with *ad libitum* access to water and feed in a 12–12 h light–dark cycle. All in vivo experiments were approved by the Purdue University Animal Care and Use Committee.

Animals were anesthetized with a ketamine (30 mg/kg) and xylazine (10 mg/kg) cocktail and the toe-withdrawal reflex was monitored for anesthetic depth. Rats were secured in an open-ended shock wave blast apparatus with a transparent plastic body covering to prevent blast exposure to the lower body. Mild bTBI was produced by a blast wave generator, which delivered a global blast pressure wave in a laboratory setting. The blast chamber consisted of a custom-built stainless-steel loading chamber and chute bolted together with a polyethylene terephthalate membrane (McMaster-Carr) sealed with an O-ring. Blast generation was achieved when pressure built up in a reservoir until it exceeded the burst strength of the diaphragm. Compressed nitrogen was used as the driver gas and was delivered to the chamber via a custom-built pneumatic switch control. The blast wave was directed downward at a distance of 50 mm from the nozzle of the blast generator to the head of the animal, with a peak pressure of 150 kPa [13]. Further details of the physical characteristics of the blast wave have been previously described [8, 13]. Sham animals (n = 4) were anesthetized accordingly and placed in the same room of the blast set-up but outside the blast wave range. Rat brains received either one blast exposure (single blast group) (n = 4) or three consecutive blast exposures (triple blast and triple blast day 3 groups) (n = 4). Rats were administered a lethal dose of ketamine/xylazine cocktail either 24 h (sham, single blast, and triple blast groups) or 72 h following blast injury (triple blast day 3 group). Rats were euthanized following the operation and perfused with chilled PBS followed by 10% neutral-buffered formalin or room temperature PBS.

### Neuropathology evaluation and immunohistochemistry

Brain samples from sacrificed rats were collected and fixed in 10% neutral buffered formalin. Tissues were processed with increasing ethanol series (70, 80, 95, and 100%), and they were treated with xylene and embedded in paraffin blocks. A Thermo HM355S microtome was used to section tissues at 5 μm thickness. Hematoxylin and eosin (H&E) staining was performed according to the standard protocol. For immunohistochemistry experiments, sections were labeled with anti-IBA-1 antibody (1:8000; Abcam, ab178847) and horseradish peroxidase-conjugated goat anti-rabbit secondary antibody (1:5000; Vector Labs, MP-745). Vector ImmPACT DAB (Vector Labs, SK-4105) was applied for 5 min, and hematoxylin counterstaining was performed. Digital images were acquired at 100X total magnification using an Olympus BX43 microscope equipped with LCmicro v2.2 software.

### Assessment of BBB pathology with immunofluorescence microscopy

Brain tissues were removed and flash frozen in a slurry of ethanol and dry ice, and embedded in OCT. Brains were sectioned with a Leica cryo-microtome at 5 μm thickness. The midbrain region was primarily evaluated because the striatum and basal ganglia regions have been correlated with BBB breakdown and permeability in AD and PD patients [13]. Tissue sections were washed with PBS and fixed in methanol. Sections were blocked with 5% normal goat serum in PBS for 45 min at room temperature, and they were incubated with primary antibodies for 16–18 h at 4 °C. Primary antibodies included anti RECA-1 (Abcam ab9774, 1:400), anti-zona occludens-1 (ZO-1) (Invitrogen 61–7300, 1:100), anti-claudin-5 (Life Technologies 34–1600, 1:50), anti-CD13 (Abcam ab108382, 1:750), anti-desmin (Abcam ab15200, 1:250), anti-platelet derived growth factor receptor β (PDGFR-β) (Abcam ab32570, 1:100), anti-Collagen IV (Millipore ab756p, 1:500), anti-glial fibrillary acidic protein (GFAP) (Millipore MAB360, 1:15,000) and anti-aquaporin-4 (AQP-4) (Millipore AB3594, 1:1000). Alexa Fluor 488 conjugated goat anti-mouse IgG, Alexa Fluor 488 conjugated goat anti-rabbit IgG, Alexa Fluor 568 conjugated goat anti-rat IgG, Alexa Fluor 568 conjugated goat anti-rabbit IgG or Alexa Fluor 687 conjugated donkey anti-mouse IgG (1:500) were used as secondary antibodies. All samples were mounted onto glass slides using DAPI containing Prolong anti-fade mounting media.

### Image analysis

Digital images were acquired via a Zeiss Axio Scope A2 at 200X or 1000X total magnification and exposure times, which were maintained between cases, for each channel were set to avoid pixel saturation and maintained between cases. Five independent midbrain region images were captured from each animal. Quantitative analysis was completed using Zen Blue software. The surface area of each BBB component was measured within an immunofluorescence image. To minimize the possibility of evaluating vascular smooth muscle cells, we restricted evaluation to capillaries measuring less than 10 µm diameter. Percent area was calculated by measuring the density of claudin-5, ZO-1, collagen IV, PDGFR-β, desmin, CD13, or aquaporin-4. The fluorescent density of these antibodies was normalized to RECA-1 area, and average values per animal were presented in the graphs. Percent stained area values (%) were provided for GFAP and RECA-1 evaluation. Raw unsaturated images were used for all quantifications. Comparisons between experimental groups were based on average values.

### Statistical analysis

Statistical analyses were performed using GraphPad Prism, version 7.03 (GraphPad Software Incorporated, La Jolla, CA). All data were analyzed using the one-way ANOVA with Tukey post hoc test. Statistical significance was set at p < 0.05 (*p < 0.05; **p < 0.01; ***p < 0.001). All graphs were developed using GraphPad Prism, version 7.03. Error bars represent standard deviation (SD).

## Results

### Delivery of the blast exposure and its effect on acute neuropathology

Briefly, an open-ended shock tube was used to deliver the blast, as previously described in other blast-induced traumatic brain injury models [8, 13]. During exposure, anesthetized animals were stabilized on a platform in order to prevent confounding head and neck movement. Any possible artifacts caused by peripheral dissemination of the shock waves were eliminated with a plastic protective body shield. In this study, a mild and nonlethal pressure (150 kPa) was chosen to deliver the shock waves. The post-evaluation characteristics are consistent with the Centers for Disease Control and Prevention and Department of Defense guidelines for mild injury [13]. Interblast time for the repetitive blast injuries was set to 5–7 min due to replacing the membrane and examination of animals. Following blast exposure, animals did not show any sign of distress or impairment, and the survival rate was 100%. Yeoh et al. showed that 150 kPa blast waves did not affect the number and volume of lesions associated with hemorrhage or blood coagulation compared to the control brains [15]. We performed a post-blast clinical examination of all 16 animals, and only one animal in the repetitive blast group demonstrated ecchymotic hemorrhage within the pinna; the hemorrhagic focus measured 2 mm in diameter. Post-blast gross examination of animals did not exhibit skull fracture, brain hemorrhage or contusion, or any visible sign of damage to parenchymal organs (kidney, lung, heart, spleen, stomach, intestines, spinal cord and liver) (data not shown).

### BBB capillaries after repetitive injury

**Fig. 1:**
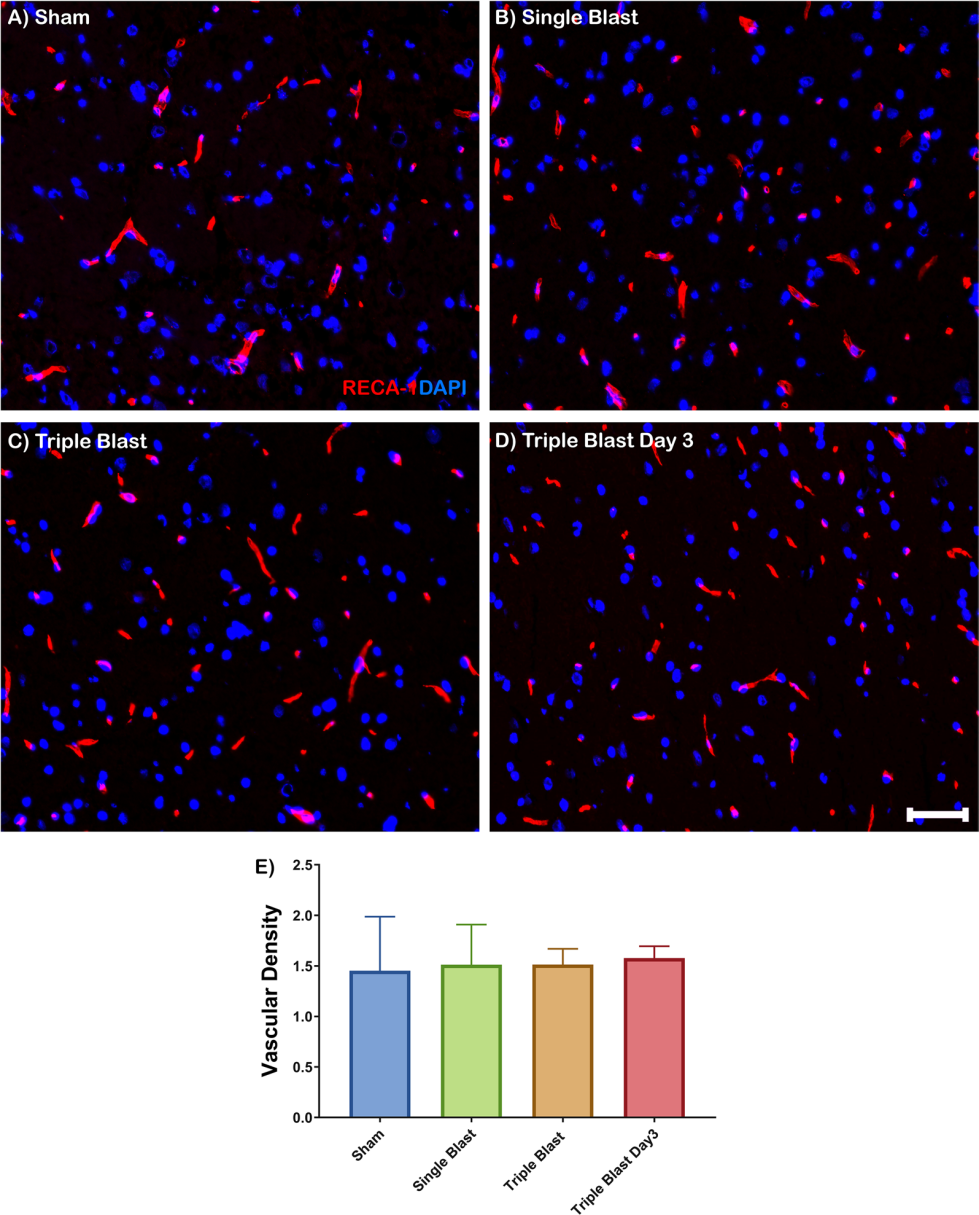
Vascular density in blast-induced traumatic brain injury. Representative immunofluorescence microscopy images of RECA-1 (red) costained with DAPI (blue) in sham (**a**), single blast (**b**), triple blast (**c**), and triple blast day 3 groups (**d**) (n = 4). **e** Quantitative analysis revealed a modest increase in RECA-1 expression pattern compared to the sham group, however the trend was not significant. Scale bar = 50 µm, *p < 0.05; **p < 0.01; ***p < 0.001, error bars show standard deviation (SD)

Brain capillaries were identified with RECA-1, an antibody specific for rat endothelial cells. RECA-1 staining showed that capillaries of the BBB were uniform and widely distributed, and highlighted by diffuse cytoplasmic RECA-1 (red) expression in sham, single and repetitive blast injuries (Fig. 1a–d). Quantitatively, there was no significant change in vascular density in single and repetitive blast injuries compared to sham control (Fig. 1e).

Structurally, endothelial cells of brain capillaries form a restrictive paracellular barrier with intercellular tight junctions to ensure the BBB homeostasis [16]. The claudin family is an integral component of the tight junction proteins of endothelial cells. Claudin-5 exclusively contributes to BBB permeability and integrity [17]. With immunofluorescence microscopy, these tight junctions were identified by a characteristic linear threadlike pattern along with endothelial cells (Additional file 1: Figure S1A-B). A similar histological pattern was observed in single and repetitive blast injury and did not alter the claudin-5 structure (green) (Additional file 1: Figure S1C–H). Quantitative analysis also confirmed that there was no significant change in claudin-5 protein expression in single and repetitive blast groups compared to the sham control (Additional file 1: Figure S1I). In the BBB, claudins are linked to the cytoskeletal system by the intercellular scaffolding proteins, zona occludens (ZO), which are essential for claudin assembly in the BBB [18]. We identified distinct ZO-1 (green) protein expression closely associated with endothelial cells (red). The structural arrangement of ZO-1 displayed a delicate linear intercellular pattern (Additional file 1: Figure S2A, B). A similar structural pattern was observed following the blast exposures (Additional file 1: Figure S2C–H). Quantitative analysis showed that ZO-1 expression did not change in single and repetitive blast exposure (Additional file 1: Figure S2I).

The non-cellular basement membrane underlies the capillary endothelial cells of the BBB. It consists of major structural extracellular matrix proteins like type IV collagens and elastin and other specialized proteins like laminins, nidogen, and fibronectins [19]. Type IV collagen is the most abundant protein in the basement membrane, and we evaluated the changes of this pan-basement membrane protein after single and multiple blast exposures (Additional file 1: Figure S3A–H). Expression of collagen IV demonstrated no significant difference following blast exposure (Additional file 1: Figure S3I).

### Repetitive blast injury is associated with increased CD13 and desmin-positive pericytes

Pericytes are located at the interface of capillary endothelial cells and are embedded within the basement membrane. Due to pericyte versatility and plasticity, we used different antibodies to identify different subpopulations of pericytes, including cell surface antigens like platelet-derived growth factor receptor β (PDGFR-β) and CD13, and intermediate filament desmin [20].

PDGFR-β is a well-characterized molecular marker of pericytes. Its activation is crucial for the proliferation and recruitment of pericytes [20]. Although changes were not significant, (Additional file 1: Figure S4), triple blast exposure slightly increased the PDGFR-β expression compared to the sham (1.17-fold, ns) and single blast (1.21-fold, ns) groups.

**Fig. 2:**
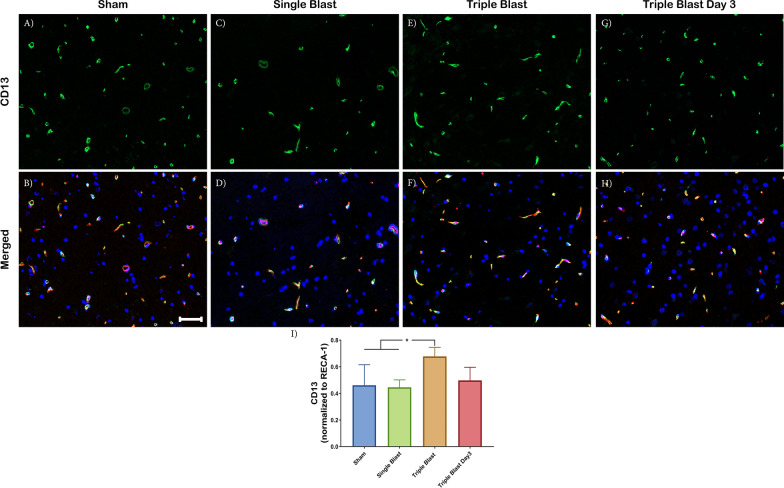
CD13-positive pericytes were elevated in repetitive mild blast-induced traumatic brain injury. Representative immunofluorescence microscopy images of CD13 (green), costained with RECA-1 (red) and DAPI (blue) in sham (**a**, **b**), single blast (**c**, **d**), triple blast (**e**, **f**) and triple blast day 3 groups (**g**, **h**) (n = 4). **i** Quantitative analysis revealed a significant increase in CD13-positive pericytes compared to the sham control and single blast injury. A recovery phenotype was observed 3 days after the repetitive injury. Scale bar = 50 µm, *p < 0.05; **p < 0.01; ***p < 0.001, Error bars show standard deviation (SD)

CD13 is a zinc-dependent membrane metalloprotease that has roles in signal transduction, differentiation, and tumor metastasis [21]. Following a single blast injury, there was no significant change in the CD13 expression (green) compared to the sham control (Fig. 2a–d, i). As a response to the triple blast exposure, CD13 expression increased compared to the single blast and sham control, 1.47-fold (p < 0.05) and 1.52-fold (p < 0.05) (Fig. 2a–f). Following the 3-day post triple blast injury, CD13 demonstrated a recovery phenotype—these findings were similar to the sham control value (Fig. 2i).

**Fig. 3:**
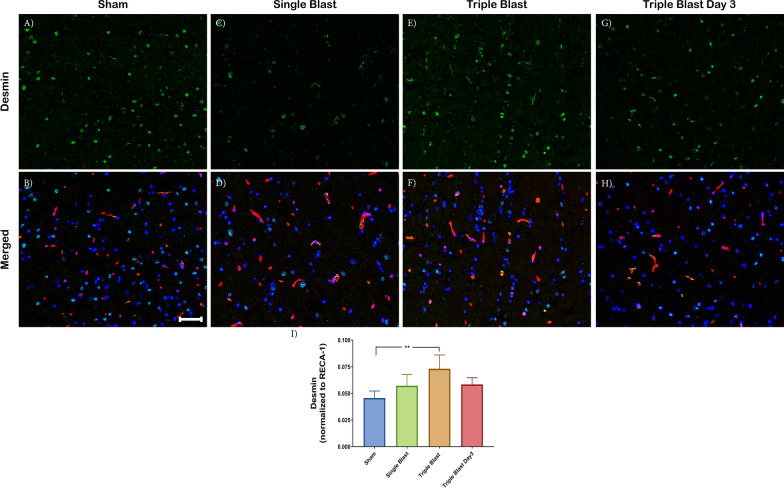
Increased desmin expression in repetitive mild blast-induced traumatic brain injury. Representative immunofluorescence microscopy images of desmin (green), costained with RECA-1 (red) and DAPI (blue) in sham (**a**, **b**), single blast (**c**, **d**), triple blast (**e**, **f**) and triple blast day 3 groups (**g**, **h**) (n = 4). **i** There was a 1.60-fold (p < 0.01) increase in desmin expression in the pericytes within the triple blast injury group. Scale bar = 50 µm, *p < 0.05; **p < 0.01; ***p < 0.001, error bars show standard deviation (SD)

We also evaluated the desmin-positive pericyte subtype (green) with immunofluorescence microscopy. Desmin is an intracellular filament protein and has a fundamental role in mechanical integrity [22]. In the single and triple blast injury groups, the desmin-positive pericyte subpopulation increased 1.25-fold (not significant) and 1.60 fold (p < 0.01) in single and triple blast compared to the sham control, respectively (Fig. 3a–f, i). Moreover, the desmin-positive subpopulation was present 3 days following triple blast injury, which was quantified as 1.28-fold (not significant) increase compared to the sham control (Fig. 3g–i). Overall, changes in CD13 and desmin-positive pericytes were most prominent in the triple blast injury group, and a recovery phenotype observed 3 days post-repetitive injury (Figs. 2, 3 and Additional file 1: Figure S4).

### Astrocytic end-feet coverage of the BBB following blast injury

Brain capillaries are covered circumferentially with astrocytic end-feet, and they contribute to BBB integrity [23]. We evaluated the astrocytic end-feet coverage with aquaporin-4 (AQP-4), glial fibrillary acidic protein (GFAP), and RECA-1 after single and repetitive blast injuries (Additional file 1: Figures S5 and S6). Yellow immunofluorescence staining indicates the co-staining of GFAP and AQP-4 (Additional file 1: Figure S6D). Polarized astrocytic end-feet control solute and water diffusion in the BBB [24]. An intermediate filament protein, GFAP, is exclusively expressed in reactive astrocytes and provides mechanical stability to astrocytic end-feet [25]. In the sham control, AQP-4 water channel proteins were detected in the astrocyte end-feet that covered capillaries (Additional file 1: Figure S6A–D). We evaluated the AQP-4 (green) expression within the RECA-1 (red) area (Additional file 1: Figure S5A–D). Quantitative analysis revealed a descending AQP-4 expression trend in the single blast (1.10-fold, not significant), triple blast (1.10-fold, not significant), and triple blast day 3 (1.16-fold, not significant) groups compared to sham control (Additional file 1: Figure S5E).

Although AQP-4 expression did not change following the single or repetitive blast injuries, high magnification images showed astrocytic end-feet displacement in some vessels despite the presence of surrounding GFAP-positive astrocytic end-feet processes (Additional file 1: Figure S6E–H, white arrow). Similarly, early signs of astrocytic end-feet displacement were detected after triple blast injuries (Additional file 1: Figure S6I–P).

### Assessment of neuroinflammation

**Fig. 4:**
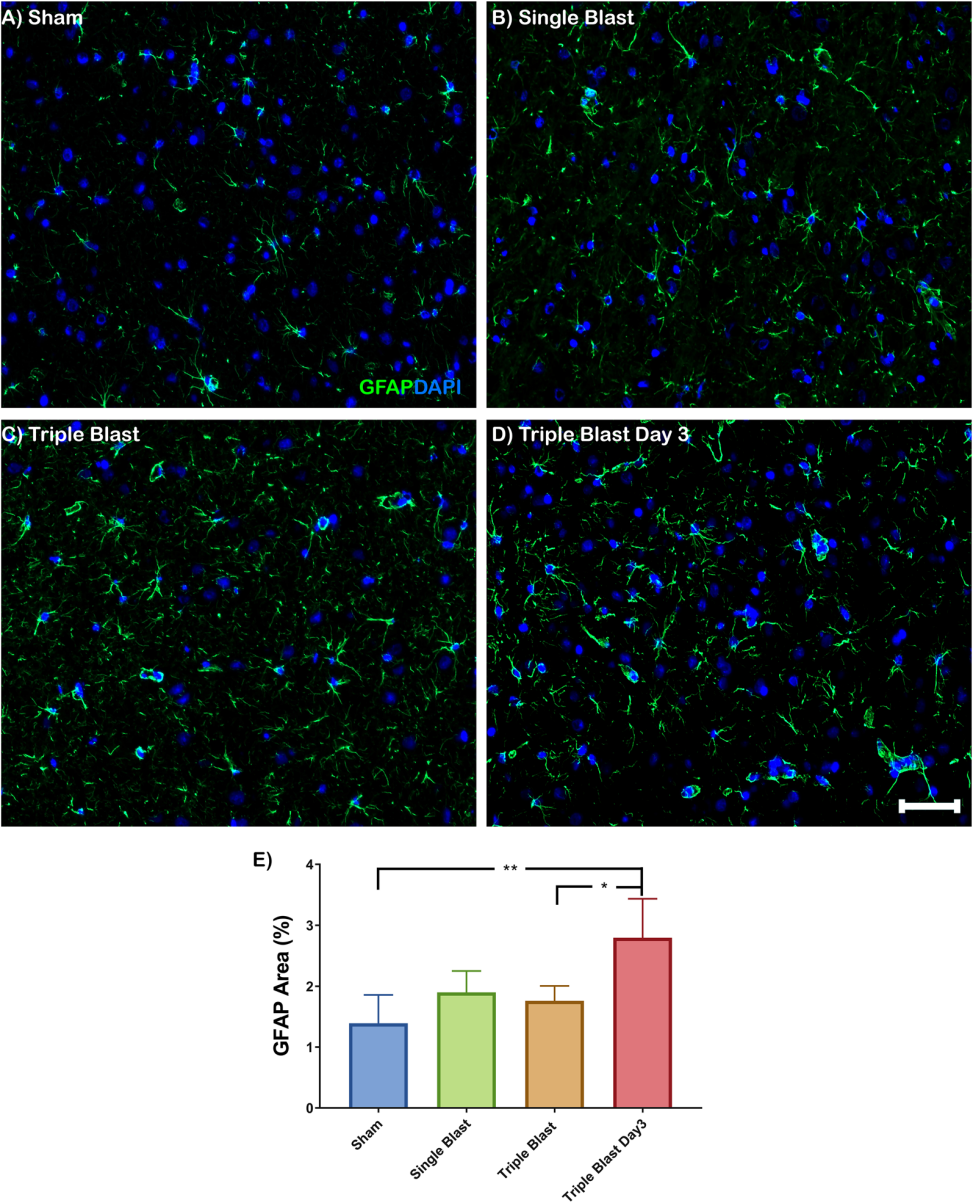
Astrogliosis is a key pathologic finding in repetitive blast-induced traumatic brain injury. Representative immunofluorescence microscopy images of GFAP (green), costained with DAPI (blue) in sham (**a**), single blast (**b**), triple blast (**c**) and triple blast day 3 groups (**d**) (n = 4). **e** Quantitative analysis showed increased GFAP expression as a long-term effect of repetitive blast injury. Scale bar = 50 µm, *p < 0.05; **p < 0.01; ***p < 0.001, error bars show standard deviation (SD)

Astrogliosis and microglial activation were identified with GFAP and Iba-1 expression, respectively (Fig. 4, Additional file 1: Figure S7). In the single and repetitive blast groups, astrocytes displayed a reactive phenotype with pronounced GFAP expression and a hypertrophic phenotype with thickening of astrocytic endfeet (Fig. 4a–d). The amount of reactive and hypertrophic astrocytes was most prominent in the triple blast day 3 group. There was a 2.01-fold (p < 0.01) increased GFAP expression in triple blast day 3 group compared to the sham (Fig. 4e). The elevated GFAP expression in triple blast day 3 group was also significantly different from the triple blast (1.59-fold, p < 0.05) group (Fig. 4e). There was an ascending trend in GFAP expression in single (1.37-fold) and triple blast (1.27-fold) groups compared to the sham control; however, they were not significant.

Ionized calcium-binding adaptor molecule 1 (Iba-1) is expressed in microglia that participate in the regulation and function of microglia [26]. In the sham control, resting microglia have highly ramified morphology with dense cell bodies and thin astrocytic processes (Additional file 1: Figure S7A). In contrast to the sham control, elongated cells with densely stained short branches and enlarged cells with larger somata, and significantly thicker processes were identified in repetitive blast injury groups. This hypertrophic morphology was also observed with some retracted secondary processes (Additional file 1: Figure S7C, D, black arrows). The bushy phenotype, which has numerous blunt and shrunken processes, was detected in the triple blast 24- and 72-h post-injury (Additional file 1: Figure S7C, D, red arrows). Moreover, a couple of amoeboid microglia characterized by large rounded somata and no branched processes were also identified (Additional file 1: Figure S7C, D, blue arrow). In the single blast group, although ramified microglia phenotype was dominant, one to two hypertrophic and amoeboid figures were identified (Additional file 1: Figure S7B).

## Discussion

The BBB is a unique microvasculature network within the neuroparenchyma, which consists of nonfenestrated endothelial cells, a complex basement membrane network, heterogeneous pericytes, and astrocytic end-feet. This tightly regulated barrier hinders the entrance of macromolecules, ions, and harmful substances to the brain [16]. BBB breakdown is the hallmark of blast-induced traumatic brain injury. Although the extent of the BBB breakdown has been investigated by measuring the BBB permeability with different imaging modalities [6, 13, 27–29], the effect of bTBI on BBB dysfunction is poorly characterized.

bTBI, also known as “invisible injury,” is a growing health concern due to the increased use of conventional or improvised-blast explosive devices. A retrospective study demonstrated that of one million veterans screened between 2007–2015, more than 10% suffered from traumatic brain injury. These data demonstrate the severity of the long-term effect of blast exposure [30]. Clinically, bTBI cases are classified as mild, moderate, and severe based on the severity of trauma [31]. It has been reported that 85% of the cases are mild injury [32]. Mild bTBI symptoms are generally transient, and neurocognitive impairment tests can be negative for those patients. Epidemiological studies have reported that mild singular blast exposure does not cause significant visible neuropathology. However, recurrence can demonstrate exacerbated pathological outcomes and behavioral abnormalities [33].

A possible correlation between BBB breakdown and bTBI has been reported in the literature [15]. Stabilization of BBB permeability following the injury can be a promising treatment strategy. There is a critical need for an understanding of the pathological changes in the BBB that leads to breakdown. The BBB pathology framework in the face of bTBI will improve targeted preventive and innovative therapies and diagnostic approaches in the early stages of the trauma.

In this study, we used an open-ended shock tube model for blast wave generation to create a clinically relevant mild blast-induced traumatic brain injury model. Animals did not show any sign of distress or impairment, and blast waves did not impact survival. Our previous work did not demonstrate any identifiable behavioral deficits in rats following injury [13]. We investigated the effect of the single blast and triple blast exposure on the BBB functional components using immunofluorescence microscopy.

The integrity of the vasculature in the brain following blast injuries is clinically important due to the triggering neuroinflammatory response and eventual degeneration. Interestingly, the single and triple blast exposure did not affect the endothelial cell morphology or vascular density (Fig. 1). Aberrant localization of tight junctions and their adaptor proteins have been demonstrated in BBB perturbations [18]. Several groups have assessed the junctional complexes of the BBB in vitro and in vivo following blast exposure. Hue et al*.* demonstrated decreased ZO-1 and claudin-5 expression along with decreased transendothelial resistance in vitro. They also observed altered ZO-1 pathology from spindle-shape to more punctate morphology [27]. In the animal model of single blast exposure, immunofluorescence and western blot analysis showed that occludin and ZO-1 were significantly increased [34]. The study conducted by Kuriakose et al*.* reported decreased occludin expression 15 min and 4 h after mild-blast injury. However, the recovery phenotype was observed 24 h after blast-injury [29].

Collagen IV is the most abundant basement membrane protein in the capillaries of the brain and crucial for the maintenance of the BBB [35]. Following single and repetitive mild-blast injury, collagen IV expression remained the same in our study (Additional file 1: Figure S3I). Changes in laminin expression after the single and repetitive blast were not evaluated due to the challenges in antibody optimization. Gama Sosa et al*.* reported abnormalities in collagen IV and laminin immunostaining following single and repetitive mild-blast (74.5 kPa) exposure [36]. The collagen IV and laminin immunostaining in perfused-fixed tissue required a proteolytic epitope unmasking step with pepsin in healthy brains. However, antibody binding following blast exposure was achieved without proteolytic digestion, suggesting potential alterations in the basement membrane structure and tightness [36]. Collagen IV has been closely associated with matrix metalloproteinase (MMP) activity in several diseases, including stroke [37]. Elevated MMP activity was demonstrated in non-blast TBI and bTBI models with significant BBB permeability [38, 39]. Nonetheless, further molecular evaluation involving other basement membrane components is essential.

Pericytes have a critical role in BBB formation and homeostasis [40]. The CNS harbors a heterogeneous population of pericytes, and those cells can be identified by their distinct morphology and notably different protein expression [41, 42]. The majority of healthy CNS vessels express PDGFR-β protein [43, 44]. Expression of other pericyte proteins is generally dependent upon the developmental stage or pathology [45–48]. Göritz et al. reported increased pericyte proliferation after spinal cord injury in a mouse model. The injured spinal cord section demonstrated a loss of CD13 expression, but PDGFR-β expression was spared. In a non-blast TBI model, Zehendner et al. demonstrated a rapid loss of PDGFR-β-positive pericytes 6–12 h post-injury; the loss was restored within 3 days [49]. Expression of desmin and NG2 on PDGFR-β-positive pericytes suggests pericyte heterogeneity in the brain following TBI [49]. In addition, Lucke-Wold et al*.* observed an increase in NG2-positive pericytes following a single blast (344 kPa) exposure [34]. Due to the heterogeneous nature of pericytes in the BBB, it remains unclear if pericytes adopt a different phenotype or a subpopulation proliferates depending on the pathology. Because we detected changes only in desmin and CD13 proteins, but not in PDGFR-β, these preliminary findings suggest that an alteration in pericyte expression as a response to injury may be the result of the differentiation of pericyte subpopulations in the BBB in the absence of proliferation. Therefore, it still remains possible that smaller differences due to antibody sensitivity were not detected. Altogether, these data indicate the importance of pericytes and their plasticity in BBB functionality as a response to the repetitive bTBI.

Astrocytes in the CNS mediate the fluid exchange and signaling between the neuroparenchyma and the BBB. Astrocyte processes extend from the astrocytic body and are highly polarized to cover 99.7% of the BBB. The organization and distribution of the water channel protein aquaporin-4 (AQP-4) in the astrocytic end-feet is determined by the degree of polarity that provides structural integrity to the BBB [50]. Although AQP-4 expression did not change following the single or repetitive blast injuries, high magnification images showed mild astrocytic end-feet displacement in some vessels despite the presence of surrounding GFAP-positive astrocytic end-feet processes (Additional file 1: Figure S6E–H, white arrow). Similarly, early signs of astrocytic end-feet displacement were detected after triple blast injuries (Additional file 1: Figure S6I–P). Also, it has been shown that the failure of the polarization leads to the disruption of the basement membrane and eventually causes vessel and neurovascular unit disintegration in a multiple sclerosis model [51]. Astrocytes are specialized cells in the CNS and one of the first responders to any insult. Typically, they preserve their domains and express scant GFAP protein. However, as a neuroinflammatory response to trauma, ischemia, or neurodegeneration, astrocytes have a hypertrophic phenotype with elevated GFAP expression [52]. Similarly, our results revealed that blast injury entails molecular and phenotypic alterations of the astrocytes. The severity of reactive astrogliosis is closely associated with the frequency of blast exposure (Fig. 4e). Also, it has been shown that hypertrophic astrocytes contribute to detrimental neuronal plasticity, which may affect the BBB pathology [53].

Activation of microglia is crucial in acute and chronic neurodegeneration and detected after brain trauma [54]. Microglial cells typically display ramified cytology that is characterized by round cell morphology with approximately 30 µm perimeter and 4–6 main branches with secondary and tertiary branches in health [55]. In primary blast injury, microglia undergo substantial phenotypic changes, called activated or reactive microglia. Activated microglia present with a hypertrophic, bushy and/or amoeboid morphology, and they are often are found in clusters [56, 57]. Glial activation characterized by rounded cell bodies and fewer ramification associated with mild-blast injury has been demonstrated [28]. These characteristics of microglia activation were identified in our preliminary findings following triple blast injury (Additional file 1: Figure S7).

**Fig. 5:**
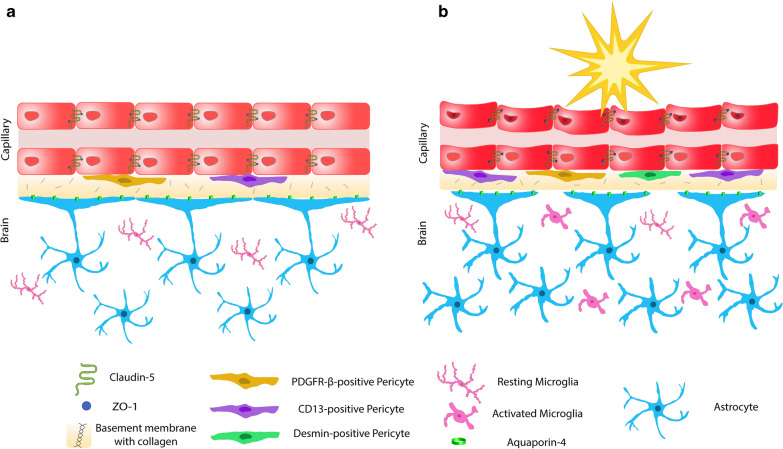
Overview of the blood–brain barrier (BBB) in health and following blast-induced traumatic brain injury. **a** Schematic representation of the brain capillaries consisting of tightly regulated endothelial cells (red), PDGFR-β (yellow) and CD13-positive (purple) pericytes embedded in collagen IV rich basement membrane (yellow–brown) with astrocytic end-feet (blue) containing aquaporin-4 water channel protein (green). Resting microglia in the neuroparenchyma are pink. **b** Following mild blast injury, there is an increase in desmin-positive (green) and CD13-positive (purple) pericytes. Astrocytic end-feet displacement is identified (astrocytic endfeet in blue and aquaporin-4 in green). GFAP expression elevates with a pronounced hypertrophic phenotype (blue). Microglia are activated and emerge with a hypertrophic, bushy and/or amoeboid morphology (pink)

Overall, pathologic findings were most striking within desmin-positive pericytes, CD13-positive pericytes, and the neuroinflammatory response in the BBB following repetitive blast injury (Fig. 5a, b). Although this study provides an analysis of the BBB in the single and repetitive mild blast injury, it has limitations. While a biologically relevant model was employed, rodents and humans have anatomical and physiological differences; therefore, their biological and restorative responses to blast injury may differ. All animals received the same blast overpressure unidirectionally due to the controlled experimental conditions, which is rare in theatre. Quantification of protein expression by immunofluorescence is dependent on the antibody specification and sensitivity. Therefore, it still remains possible that subtle differences due to antibody sensitivity were not detected.

## Conclusion

The structural and functional components of the BBB following single and repetitive blast-induced traumatic brain injury was studied in a rat model. The disruption of the BBB is a hallmark of bTBI. Our results demonstrated that repetitive blast exposure is associated with increased desmin and CD13-positive pericytes and neuroinflammation. In our model, the BBB lost the complete astrocytic end feet coverage following injury. While the therapeutic window of BBB manipulation is unclear in the face of bTBI, our results provide a promising pathologic guideline of the BBB and pave the way to improve the efficacy of therapeutic targets in neurodegenerative disease.

## Supplementary information

**Additional file 1: Figure S1.** BBB tight junctions following blast-induced traumatic brain injury. Representative immunofluorescence microscopy images of claudin-5 (green) costained with RECA-1 (red) and DAPI (blue) in sham (A-B), single blast (C-D), triple blast (E-F) and triple blast day 3 groups (G-H) (n=4). (I) There was no significant change in claudin-5 expression in the experimental groups compared to sham control. Scale bar=50 µm, * p < 0.05; ** p < 0.01; *** p < 0.001, error bars show standard deviation (SD). **Figure S2.** ZO-1 expression in repetitive blast-induced traumatic brain injury. Representative immunofluorescence microscopy images of ZO-1 (green) costained with RECA-1 (red) and DAPI (blue) in sham (A-B), single blast (C-D), triple blast (E-F) and triple blast day 3 groups (G-H) (n=4). (I) There is no significant change in ZO-1 expression compared to sham control. Scale bar=50 µm, * p < 0.05; ** p < 0.01; *** p < 0.001, error bars show standard deviation (SD). **Figure S3.** Basement membrane of the BBB following blast-induced traumatic brain injury. Representative immunofluorescence microscopy images of collagen IV (green) costained with RECA-1 (red) and DAPI (blue) in sham (A-B), single blast (C-D), triple blast (E-F) and triple blast day 3 groups (G-H) (n=4). (I) While not significant, collagen IV expression in single blast and triple blast injury was modestly diminished compared to sham control. Three days following repetitive injury, recovery of collagen IV expression was identified. Scale bar=50 µm, * p < 0.05; ** p < 0.01; *** p < 0.001, error bars show standard deviation (SD). **Figure S4.** PDGFR-β expression in blast-induced traumatic brain injury. Representative immunofluorescence microscopy images of PDGFR-β (green) costained with RECA-1 (red) and DAPI (blue) in sham (A-B), single blast (C-D), triple blast (E-F) and triple blast day 3 groups (G-H) (n=4). (I) Quantitative analysis revealed that PDGFR-β expression was slightly elevated in the triple blast group. A rescue phenotype was observed three days following bTBI. Scale bar=50 µm, * p < 0.05; ** p < 0.01; *** p < 0.001, error bars show standard deviation (SD). **Figure S5.** Aquaporin-4 expression in blast-induced traumatic brain injury. Representative immunofluorescence microscopy images of AQP4 (green) costained with RECA-1 (green) and DAPI (blue) in sham (A-B), single blast (C-D), triple blast (E-F) and triple blast day 3 groups (G-H) (n=4). (I) Quantitative analysis revealed a descending AQP-4 expression trend in the single blast (1.10-fold, not significant), triple blast (1.10-fold, not significant), and triple blast day 3 (1.16-fold, not significant) groups compared to sham control. Scale bar=50 µm, * p < 0.05; ** p < 0.01; *** p < 0.001, error bars show standard deviation (SD). **Figure S6.** Astrocyte coverage of the BBB following blast-induced traumatic brain injury. Representative immunofluorescent microscopy images of brain capillaries (red) were costained with RECA-1 (red), aquaporin-4 (green), GFAP (white) and nuclei (DAPI). Scale bar=10 µm. White arrows demonstrate astrocytic displacement. Vessels in A-H are presented in longitudinal section, and I-P are presented in cross-section. **Figure S7.** Microglial activation in repetitive blast injury. Representative immunohistochemistry images of IBA-1 (brown) in sham (A), single blast (B), triple blast (C), and triple blast day 3 groups. (A) In the sham control, microglia are predominantly ramified. (B-D) Hypertrophic (black arrows), bushy (blue arrows), and amoeboid (red arrows) morphology can be seen in response to single and repetitive blast injuries. Scale bar=50 µm.

## Data Availability

The datasets supporting the conclusions of this article are included within the article and its supplementary files.

## Abbreviations

AD: Alzheimer’s disease
AQP-4: Aquaporin-4
bTBI: Blast induced traumatic brain injury
DAB: 3,3′-Diaminobenzidine
CNS: Central nervous system
GFAP: Glial fibrillary acidic protein
Iba-1: Ionized calcium binding adaptor molecule 1
IEDs: Improvised explosive devices
MMP: Matrix metalloproteinase
PBS: Phosphate buffered saline
PD: Parkinson’s disease
PDGFR-β: Platelet-derived growth factor receptor-β
RECA-1: Rat endothelial cell antigen 1
ZO-1: Zona occludens-1
