# Supplementary material for: Structural disruption of the blood–brain barrier in repetitive primary blast injury
Source: Fluids Barriers CNS. 2021 Jan 7;18:2. doi: 10.1186/s12987-020-00231-2 (PMC7789532; doi:10.1186/s12987-020-00231-2)
Supplement: Supplementary file 1 — Additional file 1: Figure S1. BBB tight junctions following blast-induced traumatic brain injury. Representative immunofluorescence microscopy images of claudin-5 (green) costained with RECA-1 (red) and DAPI (blue) in sham (A-B), single blast (C-D), triple blast (E-F) and triple blast day 3 groups (G-H) (n=4). (I) There was no significant change in claudin-5 expression in the experimental groups compared to sham control. Scale bar=50 µm, * p < 0.05; ** p < 0.01; *** p < 0.001, error bars show standard deviation (SD). Figure S2. ZO-1 expression in repetitive blast-induced traumatic brain injury. Representative immunofluorescence microscopy images of ZO-1 (green) costained with RECA-1 (red) and DAPI (blue) in sham (A-B), single blast (C-D), triple blast (E-F) and triple blast day 3 groups (G-H) (n=4). (I) There is no significant change in ZO-1 expression compared to sham control. Scale bar=50 µm, * p < 0.05; ** p < 0.01; *** p < 0.001, error bars show standard deviation (SD). Figure S3. Basement membrane of the BBB following blast-induced traumatic brain injury. Representative immunofluorescence microscopy images of collagen IV (green) costained with RECA-1 (red) and DAPI (blue) in sham (A-B), single blast (C-D), triple blast (E-F) and triple blast day 3 groups (G-H) (n=4). (I) While not significant, collagen IV expression in single blast and triple blast injury was modestly diminished compared to sham control. Three days following repetitive injury, recovery of collagen IV expression was identified. Scale bar=50 µm, * p < 0.05; ** p < 0.01; *** p < 0.001, error bars show standard deviation (SD). Figure S4. PDGFR-β expression in blast-induced traumatic brain injury. Representative immunofluorescence microscopy images of PDGFR-β (green) costained with RECA-1 (red) and DAPI (blue) in sham (A-B), single blast (C-D), triple blast (E-F) and triple blast day 3 groups (G-H) (n=4). (I) Quantitative analysis revealed that PDGFR-β expression was slightly elevated in the triple [file 12987_2020_231_MOESM1_ESM.docx]

**SUPPLEMENTARY INFORMATION**

**Structural Disruption of the Blood-Brain Barrier in Repetitive Primary Blast Injury**

Gozde Uzunalli^1^, Seth Herr^2^, Alexandra M. Dieterly^1^, Riyi Shi^2,3^ L. Tiffany Lyle^1,4,5*^

^1^Purdue University College of Veterinary Medicine, Department of Comparative Pathobiology, West Lafayette, IN

^2^Purdue University College of Veterinary Medicine, Department of Basic Medical Sciences, West Lafayette, IN

^3^Purdue University Weldon School of Biomedical Engineering, West Lafayette, IN

^4^Center for Cancer Research, Purdue University, West Lafayette, IN

^5^Center for Comparative Translational Research, Purdue University, West Lafayette, IN

*Corresponding author


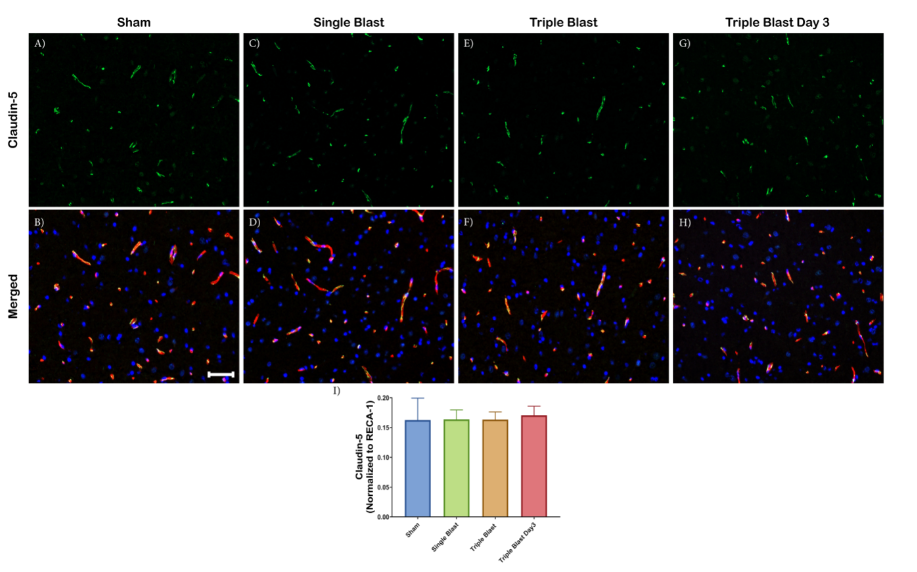


**Figure S1. BBB tight junctions following blast-induced traumatic brain injury.** Representative immunofluorescence microscopy images of claudin-5 (green) costained with RECA-1 (red) and DAPI (blue) in sham (A-B), single blast (C-D), triple blast (E-F) and triple blast day 3 groups (G-H) (n=4). (I) There was no significant change in claudin-5 expression in the experimental groups compared to sham control. Scale bar=50 µm, * p < 0.05; ** p < 0.01; *** p < 0.001, error bars show standard deviation (SD).


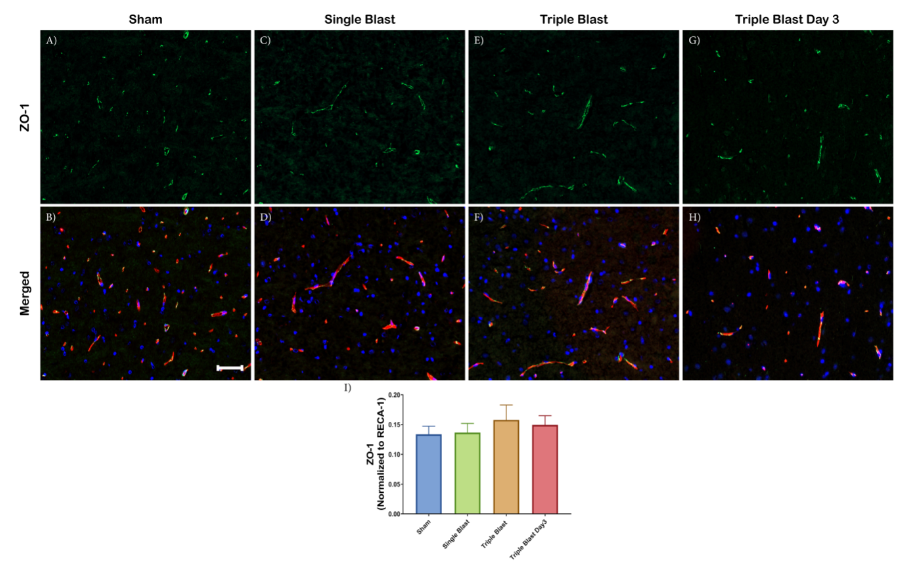


**Figure S2. ZO-1 expression in repetitive blast-induced traumatic brain injury.** Representative immunofluorescence microscopy images of ZO-1 (green) costained with RECA-1 (red) and DAPI (blue) in sham (A-B), single blast (C-D), triple blast (E-F) and triple blast day 3 groups (G-H) (n=4). (I) There is no significant change in ZO-1 expression compared to sham control. Scale bar=50 µm, * p < 0.05; ** p < 0.01; *** p < 0.001, error bars show standard deviation (SD).


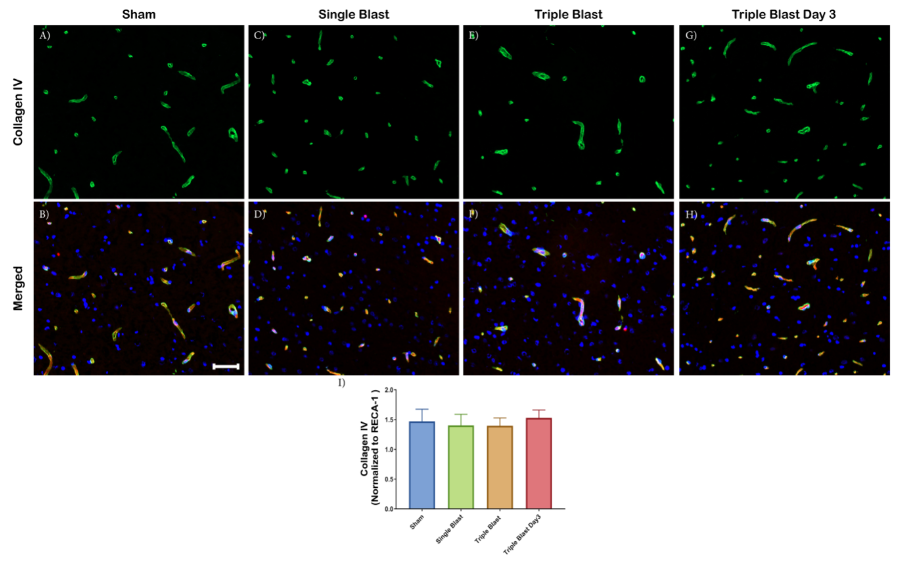


**Figure S3. Basement membrane of the BBB following blast-induced traumatic brain injury.** Representative immunofluorescence microscopy images of collagen IV (green) costained with RECA-1 (red) and DAPI (blue) in sham (A-B), single blast (C-D), triple blast (E-F) and triple blast day 3 groups (G-H) (n=4). (I) While not significant, collagen IV expression in single blast and triple blast injury was modestly diminished compared to sham control. Three days following repetitive injury, recovery of collagen IV expression was identified. Scale bar=50 µm, * p < 0.05; ** p < 0.01; *** p < 0.001, error bars show standard deviation (SD)..


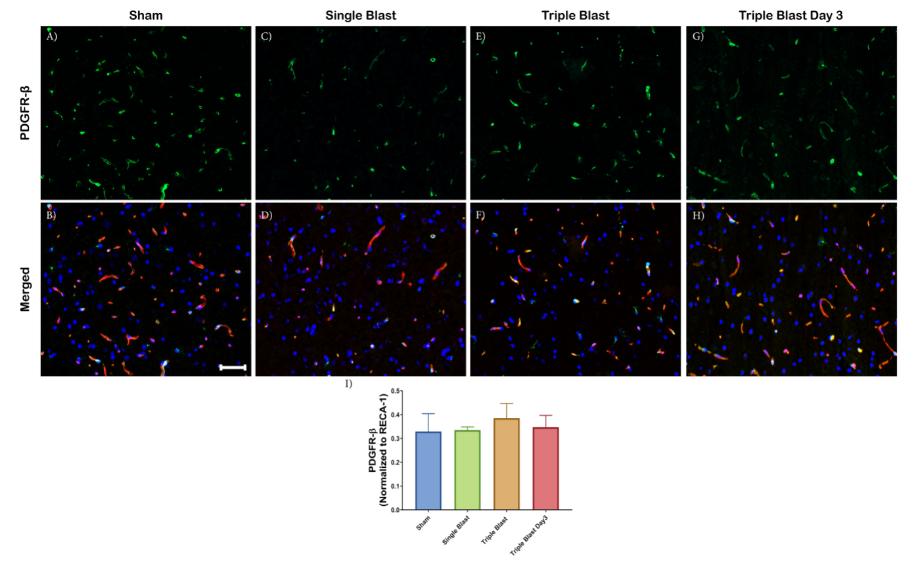


**Figure S4. PDGFR-β expression in blast-induced traumatic brain injury.** Representative immunofluorescence microscopy images of PDGFR-β (green) costained with RECA-1 (red) and DAPI (blue) in sham (A-B), single blast (C-D), triple blast (E-F) and triple blast day 3 groups (G-H) (n=4). (I) Quantitative analysis revealed that PDGFR-β expression was slightly elevated in the triple blast group. A rescue phenotype was observed three days following bTBI. Scale bar=50 µm, * p < 0.05; ** p < 0.01; *** p < 0.001, error bars show standard deviation (SD).


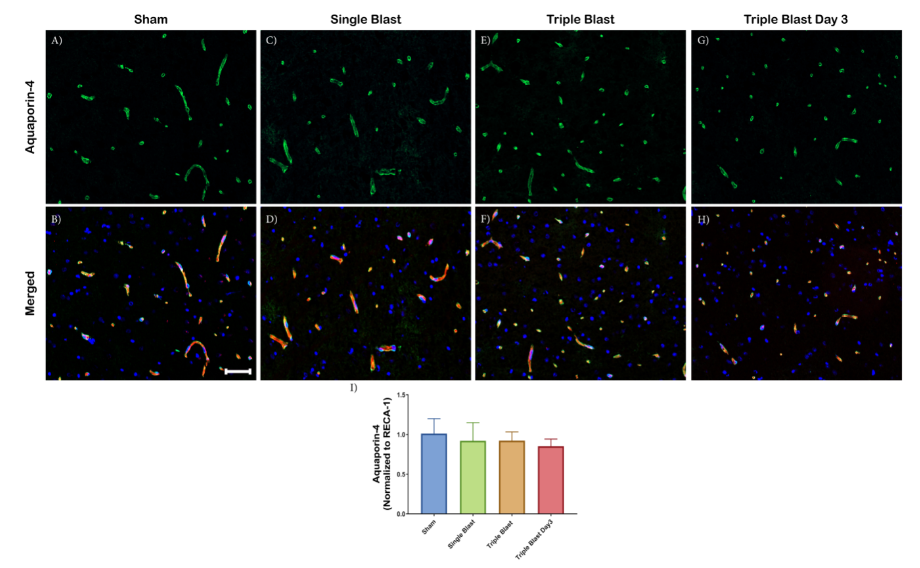


**Figure S5. Aquaporin-4 expression in** **blast-induced traumatic brain injury.** Representative immunofluorescence microscopy images of AQP4 (green) costained with RECA-1 (green) and DAPI (blue) in sham (A-B), single blast (C-D), triple blast (E-F) and triple blast day 3 groups (G-H) (n=4). (I) Quantitative analysis revealed a descending AQP-4 expression trend in the single blast (1.10-fold, not significant), triple blast (1.10-fold, not significant), and triple blast day 3 (1.16-fold, not significant) groups compared to sham control. Scale bar=50 µm, * p < 0.05; ** p < 0.01; *** p < 0.001, error bars show standard deviation (SD).


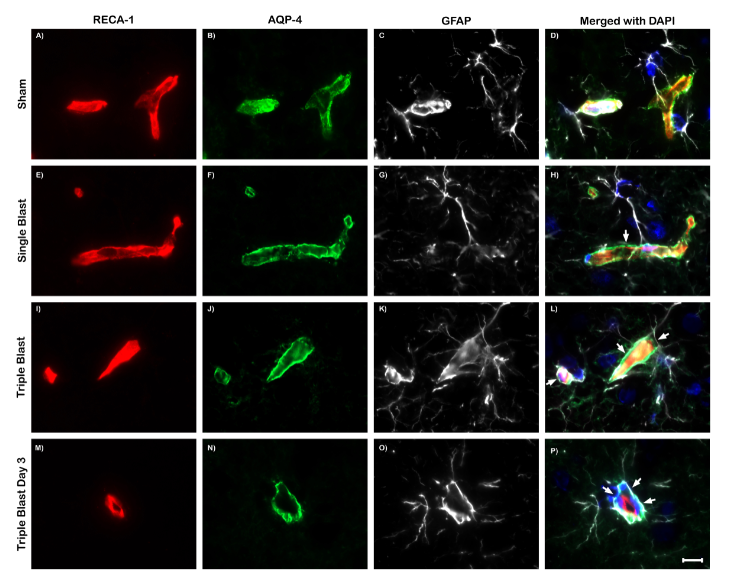


**Figure S6. Astrocyte coverage of the BBB following blast-induced traumatic brain injury.** Representative immunofluorescent microscopy images of brain capillaries (red) were costained with RECA-1 (red), aquaporin-4 (green), GFAP (white) and nuclei (DAPI). Scale bar=10 µm. White arrows demonstrate astrocytic displacement. Vessels in A-H are presented in longitudinal section, and I-P are presented in cross-section.


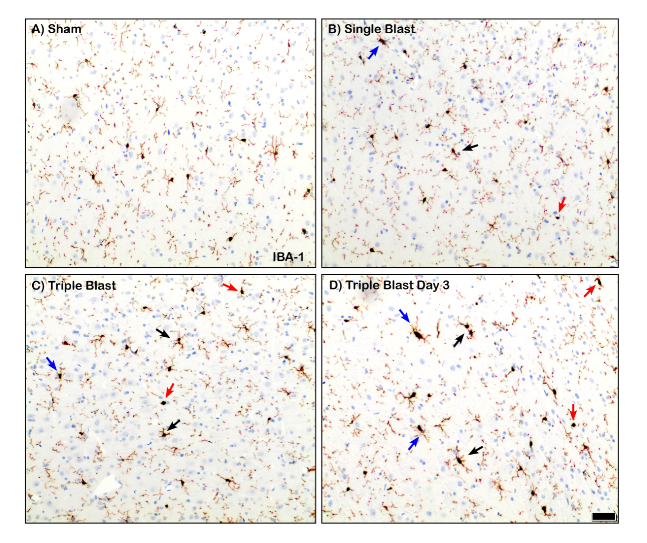


**Figure S7. Microglial activation in repetitive blast injury.** Representative immunohistochemistry images of IBA-1 (brown) in sham (A), single blast (B), triple blast (C), and triple blast day 3 groups. (A) In the sham control, microglia are predominantly ramified. (B-D) Hypertrophic (black arrows), bushy (blue arrows), and amoeboid (red arrows) morphology can be seen in response to single and repetitive blast injuries. Scale bar=50 µm.
